# Preliminary Characterization of Glass/Alumina Composite Using Laser Powder Bed Fusion (L-PBF) Additive Manufacturing

**DOI:** 10.3390/ma13092156

**Published:** 2020-05-07

**Authors:** Byeong Hoon Bae, Jeong Woo Lee, Jae Min Cha, Il-Won Kim, Hyun-Do Jung, Chang-Bun Yoon

**Affiliations:** 1 Department of Advanced Materials Engineering, Korea Polytechnic University, Siheung-si 15073, Korea; bbhqoqod@naver.com (B.H.B.); jungwoo.618@daum.net (J.W.L.); woals4102@naver.com (J.M.C.); ilwon5709@naver.com (I.-W.K.); 2 Department of BioMedical-Chemical Engineering (BMCE), The Catholic University of Korea, Bucheon 14662, Korea

**Keywords:** powder bed fusion, additive manufacturing, 3D printing, glass/alumina composite, density, mechanical property

## Abstract

Powder bed fusion (PBF) additive manufacturing (AM) is currently used to produce high-efficiency, high-density, and high-performance products for a variety of applications. However, existing AM methods are applicable only to metal materials and not to high-melting-point ceramics. Here, we develop a composite material for PBF AM by adding Al_2_O_3_ to a glass material using laser melting. Al_2_O_3_ and a black pigment are added to a synthesized glass frit for improving the composite strength and increased laser-light absorption, respectively. Our sample analysis shows that the glass melts to form a composite when the mixture is laser-irradiated. To improve the sintering density, we heat-treat the sample at 750 °C to synthesize a high-density glass frit composite. As per our X-ray diffraction (XRD) analysis to confirm the reactivity of the glass frit and Al_2_O_3_, we find that no reactions occur between glass and crystalline Al_2_O_3_. Moreover, we obtain a high sample density of ≥95% of the theoretical density. We also evaluate the composite’s mechanical properties as a function of the Al_2_O_3_ content. Our approach facilitates the manufacturing of ceramic 3D structures using glass materials through PBF AM and affords the benefits of reduced process cost, improved performance, newer functionalities, and increased value addition.

## 1. Introduction

The widely popular additive manufacturing (AM) approach based on 3D printing can be used to fabricate products with complex shapes and structures without needing a mold; in fact, some of these products cannot be fabricated with conventional molds [1,2,3]. However, 3D printing is mainly used for fabricating polymer and metal products rather than ceramics; the direct AM process cannot be applied to ceramics because they require high heat-treatment temperatures [4,5,6,7].

Meanwhile, ceramic materials are widely used in the construction, automotive, aerospace, electronics, biomedical, and chemical industries because of their excellent chemical resistance and hardness [8,9,10]. However, as ceramics have high melting points, it is difficult to manufacture complex structures using molds [11]. On the other hand, glass-ceramic materials exhibit high chemical stability, high transmittance, excellent hardness, and a relatively low melting temperature (<1400 °C); thus, they can be used to fabricate complex-shaped products by using molds and injecting glass. However, the need for complex molds increases the manufacturing costs, thereby resulting in process limitations. Moreover, as glass ceramics are brittle and easily prone to breaking, their use is restricted to products requiring high toughness. Therefore, researchers are looking to harness 3D printing technologies to fabricate complex shapes [12,13,14,15].

The typical methods of fabricating AM-based 3D ceramic shapes include the stereolithography apparatus (SLA) method [16,17,18], in which a photocurable material is mixed in ceramics, and the binder-jetting method, which injects a binder on ceramic powder [7,19,20,21,22,23,24]. Both methods require polymers, and therefore, the standard approach involves the 3D-printing of a green body containing polymers and sintering by heat treatment after the burning out of the binder [25,26]. As the process of removing polymers is essential in conventional ceramic 3D printing, it is difficult to obtain a high-density, compact-structured sintered ceramic body; hence, there is an urgent need to develop a direct AM process for ceramics [27,28].

In this study, we fabricated a sintered ceramic-composite body through direct laser melting. We developed a powder bed fusion (PBF) 3D printing process in which a mixture of Al_2_O_3_ and glass frit at a certain ratio is irradiated with a laser (L-PBF). Unlike a previous study by Datsiou et al. on L-PBF-based 3D printing using glass powder [29], here, we mixed low-melting-point glass frit and Al_2_O_3_ to improve the mechanical properties by 3D-printing the composite using L-PBF. As Al_2_O_3_ has a high melting point and does not melt with a laser, it was mixed with glass with a relatively low melting point, and 1 wt % to 3 wt % of black pigment was added to increase the absorption of laser light. Furthermore, to demonstrate the feasibility of our concept, we manufactured a scaffold by laser-irradiating the glass/Al_2_O_3_ composite and sintering at 750 °C for 20 min to produce a high-density glass/Al_2_O_3_ ceramic composite [30,31]. The resulting 70 wt % glass/30 wt % Al_2_O_3_ composite exhibited a high hardness of 578 kg/mm^2^ and density of 3.26 g/cm^3^.

## 2. Materials and Methods

Glasses based on a SiO_2_–B_2_O_3_–RO (R = Ba, Zn) ternary system were synthesized with a composite of SiO_2_ (25 mol%–40 mol%), B_2_O_3_ (25 mol%–40 mol%), and RO (R = Ba, Zn) (20 mol%–35 mol%). The glasses were prepared via conventional melt quenching at 1400 °C for 1 h. After quenching, they were ground into powders and screened to under ~50 µm in size. The powders were packed into disks with diameters of ~15 mm and heat-treated at different temperatures. The amorphousness of the glasses was measured by means of X-ray diffraction (XRD) (D2 PHASER, Bruker Corporation, Billerica, MA, USA). To determine the optimum sintering temperature of the glasses, the transition and softening points were measured by means of differential thermal analysis (DTA) (DTA-50, Shimadzu, Kyoto, Japan). To select the appropriate heat treatment temperature for the specimens, after molding the glass powder, we observed the process of glass softening at 50 °C intervals using a high-temperature microscope (HTM, Pyrotech, Seoul, Korea) as the process temperature was raised from room temperature to 800 °C at 5 °C/min. The particle size distribution was measured by using a particle size analyzer (PSA) (Mastersizer 3000, Malvern, UK). As regards the raw material sourcing, we used a commercial high-purity Al_2_O_3_ powder with D_50_ ~1 µm (>99.9%, High Purity Chemicals, Tokyo, Japan) to fabricate the glass composite, and black pigment (The Shepherd Color Company, Cincinnati, OH, USA) to increase the light absorption of the laser. To find the optimal mixing ratio, we mixed the glass and Al_2_O_3_ powders at ratios of 90:10, 80:20, 70:30, 60:40, and 50:50 wt %. Subsequently, 1 wt % to 3 wt % of the black pigment was added to the samples. The mixture was thoroughly mixed by ball-milling with alumina balls at 110 rpm for 3 h.

**Figure 1:**
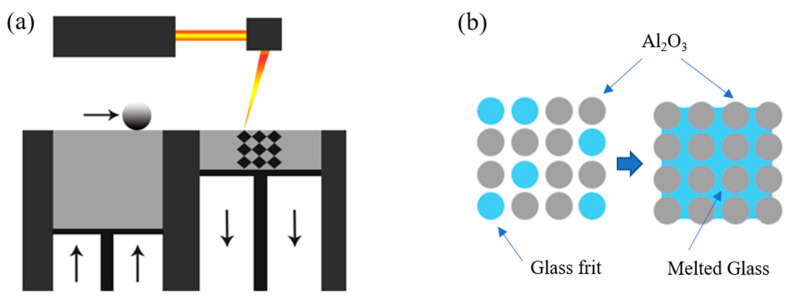
Mechanism of density improvement of glass/Al_2_O_3_ composite: (**a**) 3D printing powder bed fusion (PBF) process; and (**b**) schematic of glass frit/Al_2_O_3_ composite.

We used a 1060 nm wavelength fiber laser (FLM-I/F, LPTech, Seongnam, Korea) to irradiate the glass-Al_2_O_3_ mixture samples with a 20 W laser to produce an 80 mm model, as shown in Figure 1a. The 3D-printed shape was heat-treated at 750 °C for 20 min to improve the density. Figure 1b shows the schematic of glass frit/Al_2_O_3_ composite. To verify the surface properties, we observed the melting state of the surface using a scanning electron microscope (SEM) (Nova NanoSEM230, FEI, Hillsboro, OR, USA). In addition, the density of the sintered sample corresponding to each mixing ratio was measured by means of the Archimedes method. The reactivity of glass and Al_2_O_3_ was observed through X-ray diffraction (XRD). After sample polishing, hardness tests were conducted under a force of 2 kg for 5 s by using a Micro Vickers Hardness Tester (MVK-H200, Akashi, Tokyo, Japan). In the tests, 10 indentations were made per sample, and a total of 5 samples were used. The upper and lower bound of each hardness were calculated by the rule of mixtures combined Voigt and Reuss model, respectively [32]. According to Voigt model, the upper bound hardness, H_upper_ can be expressed as follow:

$$H_{upper}=f_{glass}H_{glass}+f_{alumina}H_{alumina},$$

where *f_glass_* and *f_alumina_* are the volume fractions of glass and Al_2_O_3_, respectively, and *H_glass_* and *H_alumina_* are the hardness of glass and Al_2_O_3_, respectively. According to Reuss model, the upper bound hardness, *H_lower_* can be also calculated as follows:

$$H_{lower}={\left(\frac{f_{glass}}{H_{glass}}+\frac{f_{alumina}}{H_{alumina}}\right)}^{-1},$$

where *H_glass_* was obtained experimentally using glass without Al_2_O_3_ and *H_alumina_* was taken from a previous study [33].

**Figure 2:**
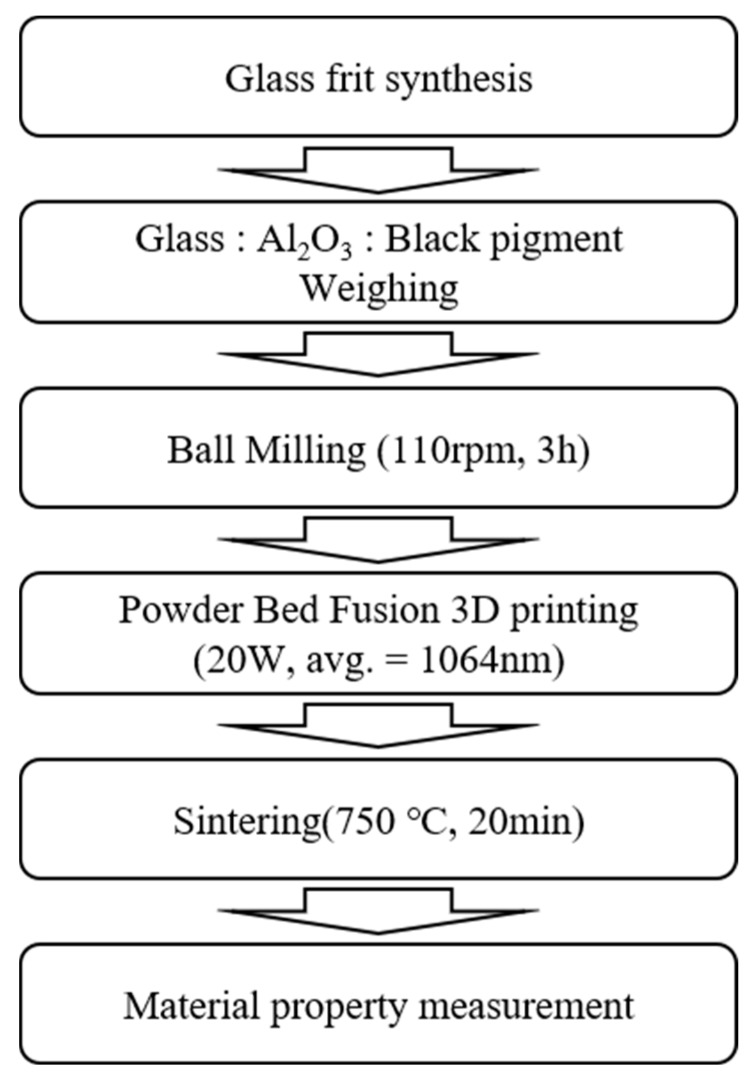
Process flow for glass/Al_2_O_3_ composite fabrication and property measurement.

To demonstrate the feasibility of our approach for manufacturing high-density products, after powder bedding, we transversely irradiated the powder with a 50-μm-diameter laser beam in a hatch pattern with a width of 500 µm and size of 50 mm to fabricate each layer. Next, after laminating the powder to 1 mm or less, the powder sample was laser-irradiated along the longitudinal direction. The above process was repeated to fabricate a continuously layered scaffold. The overall experimental processes are shown in Figure 2.

## 3. Results and Discussion

### 3.1. Results of Glass Sintering at 750 °C

**Figure 3:**
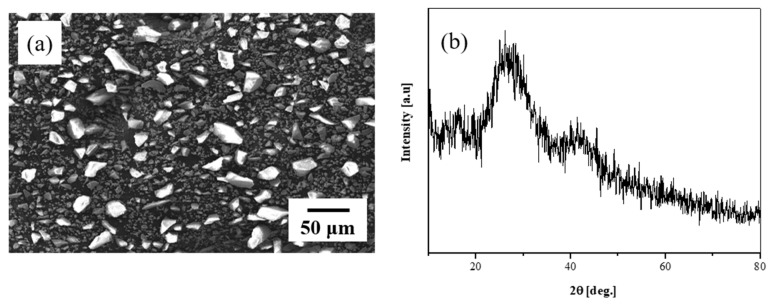
(**a**) Scanning electron microscope (SEM) image and (**b**) X-ray diffraction (XRD) results of synthesized glass frit.

The composition of the glass frit used for low-temperature sintering below 800 °C was SiO_2_–B_2_O_3_–RO (R = Ba, Zn). After melting for 1 h at 1400 °C in an alumina crucible, the glass frit was quenched in a brass mold and crushed into particles of 50 µm or smaller. Figure 3a shows an SEM photograph of the glass frit. The fabricated glass frit was quenched and crushed to particle sizes of ~10 µm to inspect the particle shape and glass phase in the crushed form. Figure 3b shows the XRD results for the crushed glass frit; the sample is clearly observed to exhibit amorphousness, with no special crystalline peaks present.

**Figure 4:**
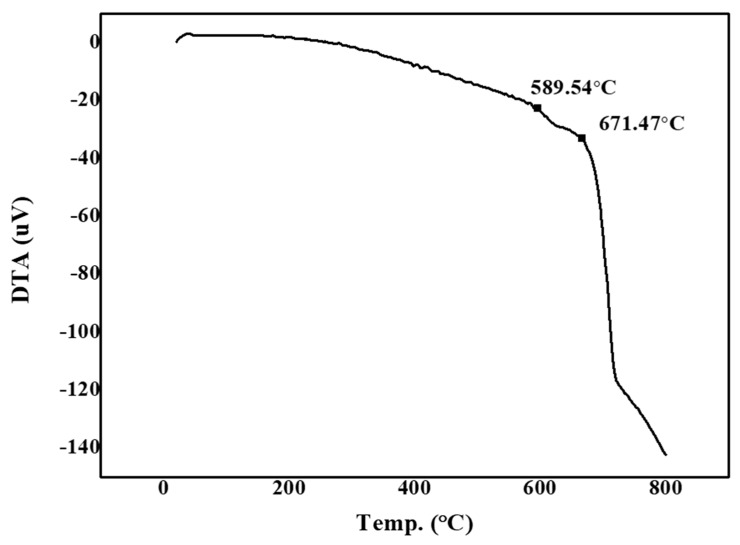
Differential thermal analysis (DTA) results of SiO_2_–B_2_O_3_–RO glass frit (R = Ba, Zn).

Figure 4 shows the measurements of the glass transition and softening points of the glass frit as per differential thermal analysis (DTA). We note that for low-temperature-sintered glass, the transition point is 589.54 °C and the softening point is 671.47 °C. No crystallization peaks are observed, which indicates that amorphous glass was synthesized.

**Figure 5:**
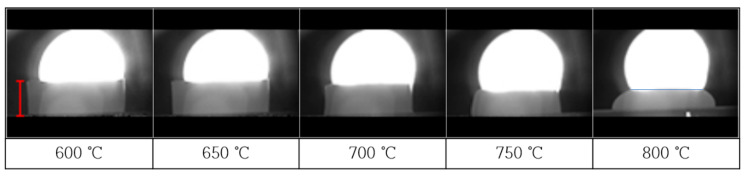
High-temperature microscope (HTM) images of SiO_2_–B_2_O_3_–RO glass frit (R = Ba, Zn) (Heating rate = 5 °C/min).

To examine the sample thermal behavior as a function of the glass temperature, we raised the temperature from room temperature to 800 °C in intervals of 5 °C/min. Figure 5 shows the corresponding high-temperature microscope images. We note that there is almost no shrinkage in the range from 600 to 650 °C. At 700 °C, shrinkage is observed to occur from the top of the sintered body to the outer vertex; a volume shrinkage of 33% and length shrinkage of 20% occurs at this temperature. At 750 °C, the frit exhibits a round-shaped spread, and a volume shrinkage of 48% (length shrinkage of 29%) is observed, which indicates that sintering is performed. At 800 °C, the volume shrinkage reaches 56% (length shrinkage of 47%), and complete melting and spreading are observed. This result demonstrates that the post-heat-treatment temperature of 750 °C is adequate to ensure sufficient sinterability of the glass frit.

Our particle-size measurements with a particle size analyzer (PSA) (Mastersizer 3000, Malvern, UK) confirmed that a homogeneous powder with d_50_ and d_max_ particle sizes of 15 and 50 µm, respectively, were obtained (Table 1). This d_50_ particle size is equivalent to that of commercial 3D printing material, and the uniformity is expected to improve when mixed with Al_2_O_3_.

### 3.2. Mixing Ratio of Fabricated Glass/Al_2_O_3_ Composite and Mechanical-Property Evaluation

**Figure 6:**
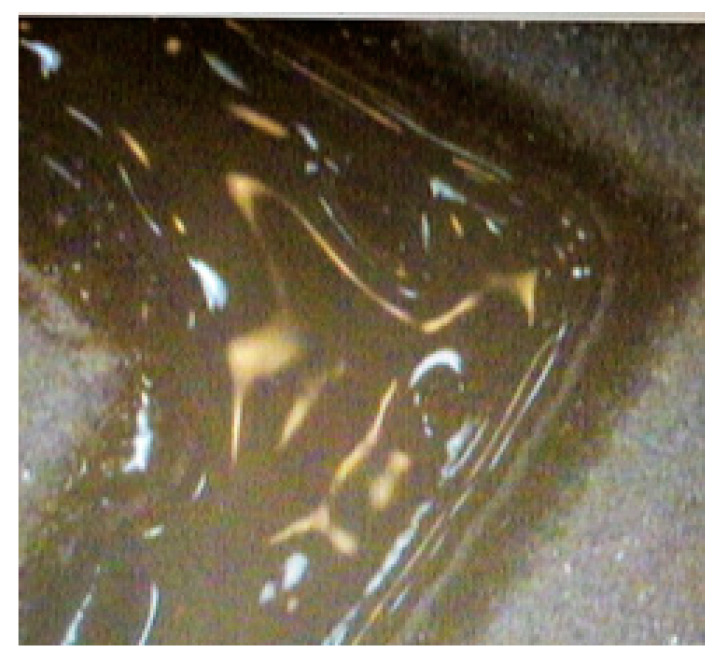
Optical microscope image of melt glass frit obtained with laser scanning.

To find the optimal composition ratio suitable for 3D printing, we considered the glass/Al_2_O_3_ mixing ratios of 60:40, 70:30, 80:20, and 90:10 wt %. After bedding, the powder was irradiated with the laser (Figure 6), and a 3D sample with dimensions of 80 mm was fabricated for each mixing ratio.

**Figure 7:**
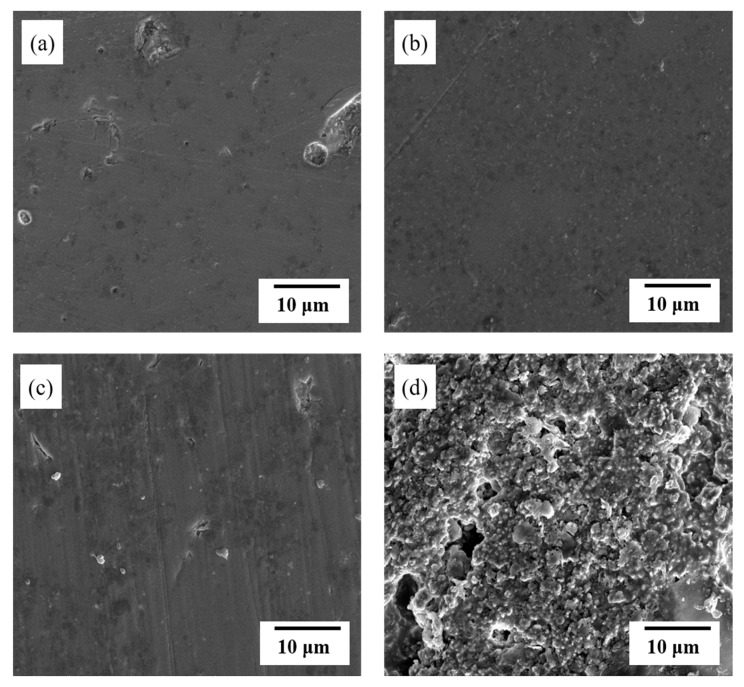
Scanning electron microscope (SEM) images of polished surface of (**a**) 90:10, (**b**) 80:20, (**c**) 70:30, and (**d**) 60:40 wt % glass/Al_2_O_3_ composite samples.

The produced shape was sintered at 750 °C for 20 min. Figure 7 shows the SEM images of the surface of the various glass/Al_2_O_3_ composites after polishing. From Figure 7a, we can clearly observe the glass-like properties of the sample due to the presence of 90 wt % glass mixed with 10 wt % Al_2_O_3_ content. Figure 7b shows the SEM image of the surface of the sample with 80 wt % glass and 20 wt % Al_2_O_3_; we can clearly observe a sintered form, wherein the glass has melted and enveloped the Al_2_O_3_ particles. As Al_2_O_3_ exhibits excellent reactivity with glass, no air bubbles are observed. In Figure 7c, corresponding to the 70:30 wt % sample, no air bubbles are observed, and the surface density is satisfactory even when the Al_2_O_3_ content of the sample is increased to 30 wt %. In Figure 7d, corresponding to the sample with a low glass content of 60 wt % and 40 wt % Al_2_O_3_, the glass does not sufficiently envelop the Al_2_O_3_ particles. This insufficient coverage results in the formation of open pores, which affords a sample with insufficient density.

**Figure 8:**
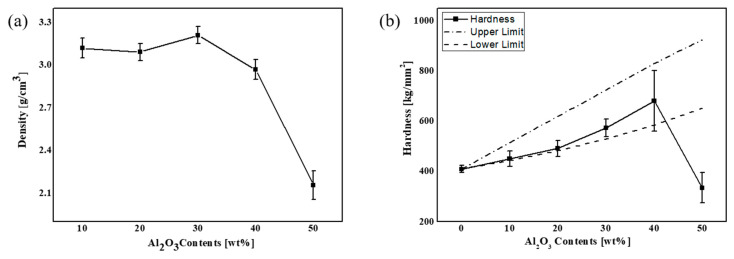
(**a**) Density and (**b**) hardness of sintered glass mixed with Al_2_O_3_ as functions of Al_2_O_3_ content.

Figure 8 shows the plots of the density and hardness of the various samples as functions of the Al_2_O_3_ content. From Figure 8a, we note that with an increase in the Al_2_O_3_ content and a decrease in the glass content, the molten glass does sufficiently envelop the Al_2_O_3_ particles, thereby leading to a density decrease. Here, we note that the densities of the glass, Al_2_O_3,_ and black pigment were 3.20 g/cm^3^, 3.95 g/cm^3^, and 5.6 g/cm^3^, respectively, and the theoretical density of the composite was obtained in proportion to the lever rule. The calculated theoretical and experimental densities are listed in Table 2. We note that the overall density remains high at ~95.3% of the theoretical density of 3.42 g/cm^3^ for the Al_2_O_3_ range of 10% to 30%, and the density significantly reduces when the Al_2_O_3_ content increases to 40%; this result corresponds to the SEM images in Figure 7. Therefore, the efficiency of 3D printing can be maximized by setting the Al_2_O_3_ content to 30%, which yields the highest density (95.3%). Meanwhile, from Figure 8b, we note that the hardness increases with increase in the Al_2_O_3_ content; the hardness increases from 452 HV for the Al_2_O_3_ content of 10% to 772 HV for the Al_2_O_3_ content of 40%, and subsequently it decreases to 337 HV for the Al_2_O_3_ content of 50%. This is because there is an insufficient amount of glass available to envelop the Al_2_O_3_ in the glass/Al_2_O_3_ composite as the Al_2_O_3_ content increases. The experimentally obtained results were closer to the estimated lower bound, which is thought to be related to insufficient densities of the experimental samples. The average hardness values for the composite with Al_2_O_3_ ranging from 10% to 40% were within the upper and lower bounds but the value for 50% was out of the estimated bound, which can be explained from the relatively low experimental density. Therefore, from Figure 7a,b and Figure 8, we can confirm that the Al_2_O_3_ content of 30% is the most suitable for 3D printing.

**Figure 9:**
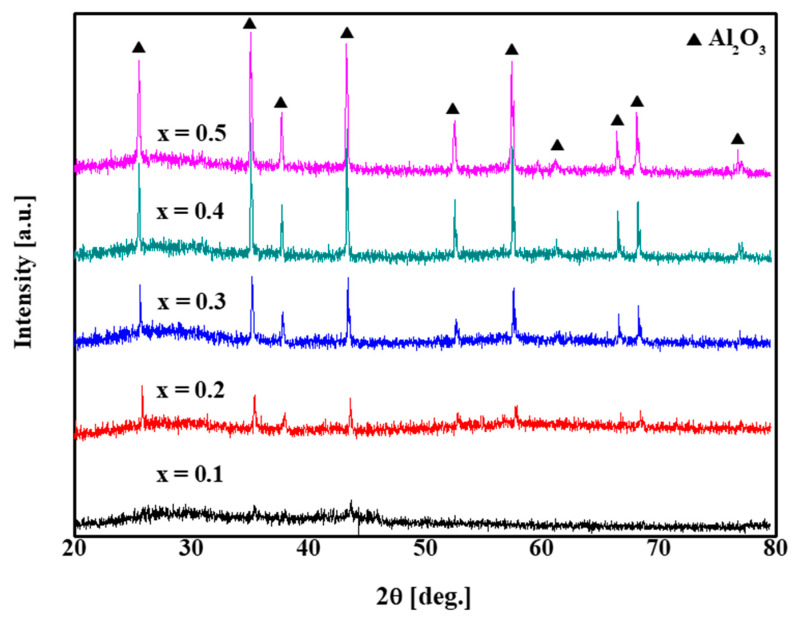
X-ray diffraction (XRD) results of [xglass/(1 − x)Al_2_O_3_)] composite samples for x = 0.1 − 0.5 Al_2_O_3_.

Figure 9 shows the XRD analysis results of the glass/Al_2_O_3_ composites. For 10 wt % Al_2_O_3_, the amorphous phase is most apparent, although Al_2_O_3_ peaks are also observed. With increase in the Al_2_O_3_ content from 20 wt % to 50 wt %, the number of crystalline Al_2_O_3_ peaks increases. This result confirms that no secondary phase is generated by reactions between the amorphous glass and crystalline Al_2_O_3_.

**Figure 10:**
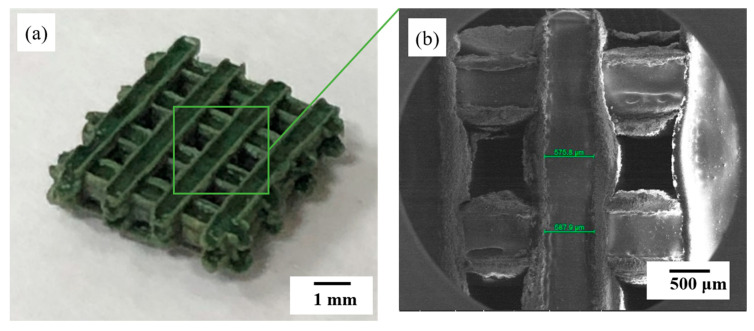
(**a**) Optical and (**b**) scanning electron microscope (SEM) images of a 3D scaffold manufactured by powder bed fusion (PBF).

Figure 10 shows the image of a scaffold constructed using the glass/Al_2_O_3_ composite with 30% Al_2_O_3_. Figure 10a shows the scaffold image after 50 µm laser irradiation in a hatch pattern. In this phase of the study, laser irradiation was performed along the transverse direction after powder bedding. The platform holding the scaffold was next lowered along the Z-axis, the powder was bedded to 1 mm or less, and the laser was used to irradiate the powder layer along the longitudinal direction. This process was continuously repeated. Each line was about 500 µm in length, and after several layering processes, a continuous layered scaffold with a size of ~5 mm was fabricated. Printing was performed as the glass frit melted and enveloped the Al_2_O_3_. The sample with 70:30 wt % of glass:Al_2_O_3_ was used in this case. Figure 10b shows the highly dense scaffold fabricated after heat treatment at 750 °C. A 3D shape was fabricated out of this ceramic material by laser-irradiating only the powder with no binder. As there was no debinding process involved, it was possible to produce a complex 3D structure with high density.

## 4. Conclusions

In this study, we fabricated a glass/Al_2_O_3_ composite using powder bed fusion (PBF) 3D printing. Laser 3D printing, which is generally used for metals and more recently tested with glass, was applied to a ceramic material to fabricate a composite molded body without polymer binder. The fabricated glass frit (particle size d_max_ < 50 µm) exhibited no crystallinity as per our XRD analysis. The frit was subsequently mixed with commercial Al_2_O_3_ at ratios ranging from 90:10 wt % to 50:50 wt %, with 1 wt % to 3 wt % black pigment being added for enhancing laser-light absorption. After fabricating the molded body by laser-irradiating the powder at each mixing ratio, we measured the resulting hardness. A subsequent XRD analysis confirmed that the glass and crystalline Al_2_O_3_ phases did not react with each other. The composite with glass:Al_2_O_3_ = 70:30 wt % exhibited a hardness of 578 kg/mm^2^ and density of 3.26 g/cm^3^; this ratio was determined as the optimum mixing ratio. This composite sample was irradiated by a 20 W, 1060 nm wavelength fiber laser (MLPS-20, Germany) to obtain a sintered composite. To apply the PBF method and demonstrate the practicability of using the composite, after bedding the glass:Al_2_O_3_ = 70:30 wt % powder, we transversely irradiated the sample with a 50-μm-diameter laser beam in a hatch pattern with a width of 500 µm and size of 50 mm to fabricate one layer. Next, after lamination to 1 mm or less, the powder was irradiated by the laser along the longitudinal direction. The above process was repeated to fabricate a high-density continuously layered glass/Al_2_O_3_ composite 3D scaffold with a size of 80 mm. The successful fabrication of this scaffold indicates the practical applicability of our approach to manufacture high-density glass/Al_2_O_3_-composite-based products.

## Figures and Tables

**Table 1:** Thermal properties and particle sizes of developed glass.

Classification	Unit	Value
Transition Temperature	°C	589
Softening Temperature	°C	671
Particle Size (d_50_)	µm	15
Particle Size (d_max_)	µm	50

**Table 2:** Theoretical and experimental densities of composites.

Al_2_O_3_ Content (wt % (vol %))	Theoretical Density of Composite (g/cm^3^)	Experimental Density (% of Theoretical Density)
10 (08)	3.28	94.8
20 (17)	3.35	92.5
30 (25)	3.42	95.3
40 (34)	3.49	85.1
50 (44)	3.56	60.7
